# Macrocytic Anemia and Thrombocytopenia Induced by Orlistat

**DOI:** 10.5812/ijem.6721

**Published:** 2013-10-01

**Authors:** David Palacios-Martinez, Juan Carlos Garcia-Alvarez, Nieves Montero-Santamaria, Olga Patricia Villar-Ruiz, Antonio Ruiz-Garcia, Raquel Asuncion Diaz-Alonso

**Affiliations:** 1 San Blas Health Center, Primary Health Care, SERMAS- Madrid Health Service, Parla, Madrid, Spain; 2 Local Office of Serranillos del Valle, Primary Health Care, SERMAS- Madrid Health Service, Griñón, Madrid, Spain; 3 Service of Obstetrics and Gynecology, 12th October Universitary Hospital , SERMAS- Madrid Health Service, Madrid, Spain; 4 Pinto Health Center, Primary Health Care: SERMAS- Madrid Health Service, Pinto, Madrid, Spain

**Keywords:** Anti-Obesity Agents, Obesity, Obesity, Morbid, Thrombocytopenia

## Abstract

**Introduction:**

The overall incidence of obesity and its prevalence is increasing continuously. The obesity is a cardiovascular risk factor whose importance is increasing too. It is associated with many chronic conditions such as type II diabetes mellitus or cardiovascular diseases. The obesity is also implicated as a risk factor for several kinds of cancer such as esophagus, pancreas, colon, rectum, breast cancer in menopausal women. The treatment of the obesity may reduce the incidence of these diseases. The mainstray of the treatment of obesity is changing the lifestyles, but obesity´s treatment may need drug therapy or even though surgical treatment.

Orlistat is a specific inhibitor of gastrointestinal lipases, which stops fat absortion. It is used along with a hypocaloric diet, for obesity´s treatment. The beneficial effects of orlistat include weight loss, the improvement of blood pressure´s control, it may delay the development of diabetes mellitus, and it may reduce HbA1c.

**Case Report:**

Besides the interaction with other drugs (mainly warfarin and amiodarone).

Orlistat´s mainly side effects are gastrointestinal disorders such as the existence of oily spotting from the rectum, abdominal pain or discomfort, fecal urgency. There are also side effects at other levels, like flu symptoms, hypoglycemia, heathache or upper respiratory infections. There are other side effects with very low incidence but clinically relevant like pancreatitis, subacute liver failure, severe liver disease, myopathy, or tubular necrosis secondary to oxalate nephropathy induced by Orlistat.

**Discussion:**

In this case report appears a new adverse effect of Orlistat that has not been described above: thrombopenia and macrocytic anemia.

## 1. Introduction

Considering causality, the new adverse effects of marketed drugs could be classified as certain, probable, possible, unlikely, unclassified or unassessable. Through this case report, we will present a new probable, rare and unusual adverse effect of Orlistat.

The overall incidence of obesity and its prevalence is increasing continuously (1). Obesity is associated with many chronic conditions such as type 2 diabetes mellitus (T2DM) or cardiovascular diseases. Obesity´s importance as a cardiovascular risk factor is increasing. It is also implicated as a risk factor for certain types of cancer: adenocarcinoma of the esophagus, pancreas, colon, rectum, breast cancer in menopausal women (although it is a protective factor in premenopausal women), endometrial cancer, renal cancer, bladder and other cancers such as prostate cancer (where it is mainly related to high-grade tumors). 

Obesity´s mainstay of treatment is changing the lifestyles. Small changes in these styles have a modest but significant effect on weight loss. These modifications include a reduced calories diet along with physical exercise and psychosocial interventions continued (1, 2). Drug therapy is indicated when body-mass-index (BMI) > 30 kg/m^2^ or BMI > 27 kg/m^2^ with comorbidity. Surgical treatment is indicated where BMI > 40 kg/m^2^ or BMI > 35 kg/m^2^ with associated diseases provided that other treatment options have failed (2, 3).

Orlistat is a potent, specific and long action inhibitor of gastrointestinal lipases (4). It exerts its therapeutic action in the stomach´s lumen and small intestine by forming a covalent bond with the active serine site of gastric and pancreatic lipases, thus the inactivated enzyme is not able to hydrolyze dietary fat absorbable components (1, 3, 4). It is used in conjunction with a hypocaloric diet for the treatment of obesity and weight maintenance by preventing the absorption of fat in the intestine (1-3). Orlistat (Xenical^®^, Alli^®^) is approved for obesity´s treatment in Spain since 1999. It can be dispensed without prescription or any medical control on the form of lower doses (Alli^®^ 60mg Orlistat).

During our daily clinical practice, we have seen a side effect of this drug not previously described: anemia, thrombocytopenia and macrocytosis.

## 2. Case Report

A 34 -years-old-woman allergic to Cloxacillin sodium, with family history of diabetes mellitus a mother, grandmother and uncles and acute myocardial infarction in man. Not hypertensive or diabetic or dyslipidemic. Smoker of 20 cigarettes per day, with no other toxic habits Had chronic hidradenitis. 

On December 2009, she was sent from our Primary Care Consultation to Service of Endocrinology, Infanta Cristina Hospital, due to morbid obesity without compression or thyroid dysfunction or adrenal. Physical exams: weight 97.7 kg, height 152 cm, BMI 42.3 kg/m ^2^, blood pressure 122/68 mmHg, abdominal perimeter 113 cm, we did not find other abnormalities except pearly streaks on abdomen and lower limb venous insufficiency. She presented a normal analytical *(Table 1) *. She was diagnosed with grade III obesity, and proposed for bariatric surgery at Surgery Service. There, she was sent to the Psychiatry Service for evaluation. This evaluation did not appreciate psychopathology or other circumstances that contraindicated the inclusion of the patient in the bariatric surgery program. Therefore, she started dietary treatment, ruled 1500 calories diet, regular exercise and she was treated with Orlistat 120 mg twice daily. She was quoted for review with analytical results in endocrinology clinic in March 2010. 

**Table 1.** Analytical Developments

	Orlistat (12-17-2009)	Orlistat and Doxycycline (3-5-2010)		NO Orlistat, NO Doxycycline (3-29-2010)		
	11-03-2009	03-01-2010	03-17-2010	04-27-2010	05-01-2010	09-06-2010
**Leukocytes , µL**	12.900	12.120	12.850	10.640	9.960	10.110
**Neutrophils , µL**	8.100	9.000	9.000	7.200	6.800	6.400
**Lymphocytes , µL**	4.100	2.600	3.000	3.000	2.700	3.000
**Monocytes , µL**	400	400	400	300	300	400
**Erythrocytes, µL**	4.180.000	3.310.000	2.320.000	3.810.000	3.800.000	4.198.000
**Hemoglobin, g/dL**	13.1	10.3	8	12.1	12.3	13.5
**Hematocrit, %**	37.3	32	24.4	38.6	38.5	40.7
**MCV, fL**	89.2	93.6	105.2	101.4	101.3	92.4
**Platelets, µL**	310.000	60.000	58.000	167.000	1.800.000	285.000
**R.N.I.**		1.09	1.08		0.92	
**Prothrombin time, sec**	12.5	12.5				
**APTT, sec**		25,6	24		22.4	
**Fibrinogen, mg/dL**		493.4	411.6		>500	
**Antithrombin 3**			101.6 %			
**Glucose, mg/dL**	88	95			89	
**Creatinine, mg/dL**	0.7	1			0.	
**Cholesterol, mg/dL**	163	143			131	
**Chol-HDL, mg/dL**	34	32				
**Chol- LDL, mg/dL**	101	84				
**Triglycerides, mg/dL**	140	133			101	
**AST, U/L**	13	16			14	
**ALT, U/L**	15	15			15	
**LDH, U/L**				138	153	
**Bilirrubin, mg/dL**				0.4	0.2	
**Iron, mg/dL**		81			42	
**Ferritin, ng/mL**		427			149	
**Vit B12, pg/mL**		318			379	
**Folic Acid, ng/mL**		3			4.9	
**C reactive protein, mg/L**					12.4	
**Rheumatoid Factor, UI/mL**					6.9	
**Ig G, mg/dL**					1575	
**Ig M, mg/dL**					63	
**Ig A, mg/dL**					299	
**Haptoblogin**					190	
**β2 microglobulin, mg/L**					2.3	
**HBs Ag**					Negative	
**Anti HCV Ab.**					Negative	
**Anti HIV Ab.**					Negative	
**TSH, µU/mL**	2.45	2				
**Blood smear**			Normal, no blasts		Normal, no blasts	Normal, no blasts

The patient came to our Primary Care office on 03/05/2010 due to spontaneous bruising and fatigue from a month and a half of evolution. She had closely followed the treatment and lost weight (92.7 kg, BMI 40.1 kg/m^2^). Her examination revealed a questionable splenomegaly and widespread bruising in various stages of resolution. It was requested analytical, extracted on 03/17/2010. On that same visit, she was treated with Doxycycline 100mg per day due to an episode of hidradenitis. 

On 03/29/2010, she came to collect these analytical results, which showed Leukocytes 12,850 x 10 ^3 ^/mL, normal formula, Hemoglobin 8 mg/dL with mean corpuscular volume of 105.2, hematocrit 24.4% and platelets 58000 x 10 ^3 ^/µl, and normal levels of cobalamin and folic acid. These findings were confirmed by the analytical extracted on requested by endocrinologists before starting treatment with doxycicline *(Table 1). *

Chest x-ray was claimed (it was normal) and she was derived to Hematology Department of Infanta Cristina Hospital to study for the results and the possibility that it was secondary to hematological processes as aplastic anemia (although the presence of splenomegaly would be against this diagnosis), Myelodysplastic Syndrome, Myeloproliferative Syndromes (myelofibrosis), hypersplenism, idiopathic thrombocytopenic purpura, etc. Meanwhile medication was discontinued, suspecting that this finding could be secondary to drugs: Orlistat was launched in December 2009, and Doxycycline started in March 2010. 

## 3. Evolution

She was valued for the first time in Hematology Outpatients on , 27 days after stopping the medication. They requested an urgent analytical that same day *(Table 1): *the direct Coombs test was negative, the seric protein and immunogloblulins analysis was normal, the bicytopenia was resolved and the blood smears detected no blasts. Thereby, she was cited for possible drug toxicity in Hematology with full analytical results. 

 According to data sheet, Orlistat does not produce laboratory abnormalities. Although, doxycycline may, but there was no temporal coincidence. 

Following analysis of May 2010 (Table 1), the hematological abnormalities were resolved, persisting macrocytosis which was corrected in September 2010. Hematology finally diagnosed the patient with thrombocytopenia and macrocytosis secondary to Orlistat, which resolved after discontinuation of treatment. Such effect was officially reported as drug reactions to the Madrid Health Service. 

## 4. Discussion

Weight loss and thereby achieving better control of blood pressure have been described as beneficial effects associated with Orlistat (5). Several studies have demonstrated the efficacy of Orlistat versus placebo in weight loss and its maintenance (2, 4, 5). It is shown that Orlistat with dietary interventions has beneficial effects on cardiovascular risk factors by decreasing LDL-levels, blood pressure and glucose. These beneficial effects are increased with de combined use of L-carnitine primarily in diabetic patients (4). Several studies suggest that weight loss achieved with Orlistat delay the development of T2DM (at least during the study period). Nevertheless, it is unknown whether this finding is translated or not long-term clinical benefits (5). Several clinical trials show a significant reduction in HbA1c in poorly controlled diabetic patients treated with Orlistat. Nevertheless, they have failed to establish if the effect on HbA1c is independent of weight loss (2). 

Orlistat interferes with the absortion of many other drugs such as warfarin, amiodarone, cyclosporine, thyroxine, fat-soluble vitamins, thyroxin, etc (6). 

Orlistat has several side effects due to enzyme inhibition:

The most frequent and specific are gastrointestinal: oily spotting from the rectum, abdominal pain or discomfort, bloating with fecal discharge, fecal urgency, fatty or oily stools, loose stools and increased defecation.

Less common disorders are fecal incontinence and abdominal disruption (2, 7).

Other side effects with high incidence (> 10%) are flu symptoms, hypoglycemia, headache and upper respiratory infections. Anxiety, irregular menstrual cycle, urinary tract infections and fatigue may occur frequently but with an incidence < 10% (1).

Other side effects have been described with lower incidence but clinically relevant: there are few reported cases of pancreatitis with and without elevated amylase, subacute liver failure, sever liver disease, cholestatic hepatitis, tubular necrosis secondary to oxalate nephropathy induced by Orlistat, myopathy and a case of hepatocelullar necrosis causing mass death running as rare idiosyncratic drug reaction (8-13). To our knowledge, there are no reported cases of macrocytic anemia and thrombocytopenia due to Orlistat prior to this one. Several factors could be involved in our patient´s bicytopenia. We will discuss the main of them:

### 4.1. Doxycycline

**Figure 1.**
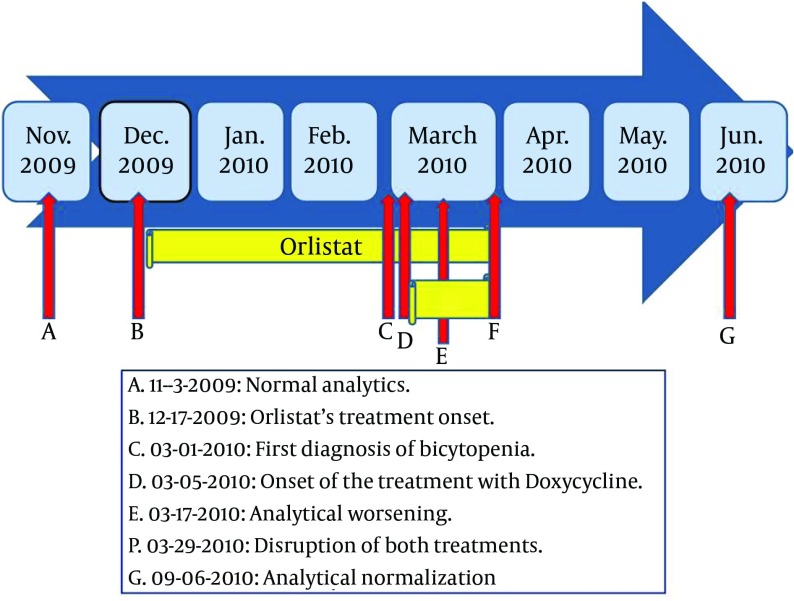
Time Sequence

It is the safest tetracycline ( 14 ). Hematologic side effects of tetracyclines are rare ( 14 ). There have been described hemolytic anemia, thrombocytopenia, neutropenia and eosinophilia ( 14 ). In our case, our patient presented macrocytic anemia before starting the treatment with Doxycycline (Figure 1). Therefore, we ruled out doxycycline as a cause of her bicytopenia. 

### 4.2. Macrocytosis

Several etiologic factors could be involved in the development of macrocytosis, being mainly alcoholism, liver diseases (particularly when caused by alcohol), interference in the biosynthesis of DNA, myelodysplastic syndromes, hypothyroidism, etc. The interference in the biosynthesis of DNA may be due to deficiency in folate and cobalamine (such as poor diet or malabsorption), after the treatment with drugs which inhibit the absorption of cobalamin and folate in the intestine, or which inhibit several enzymes necessary for the biosynthesis of DNA Hydroxyurea, Zidovudine, Cytosine arabinoside, Methrotexate, Azathioprine or 6-mercaptopurine, Cladribine, Capecitabine, Imatinib, Sunitinib (15, 16). Nevertheless, the causes of macrocytosis were ruled out in our patient by history and the appropriate tests.

### 4.3. Thrombocytopenia

Acute, severe and symptomatic thrombocytopenia is the most common presentation of thrombocytopenia due to drugs. The mechanism involved is often due to drug-dependent antibodies. They are formed against a novel antigen on the platelet surface, which is created by the binding of the drug to a protein of the platelet surface. There are two different mechanisms:

The most common mechanism of thrombocytopenia due to drug-dependent antibodies is the increased destruction of platelets. These drug-dependent antibodies bind to platelets through their Fab regions, and through several glicoproteins (GP Ib/V/IX, GP IIb/IIIa, V GP) and through platelets endothelial cellular adhesion molecule I (PECAM-1) (17, 18).

Decreased production of platelets by megakaryocytes. It may contribute to the drug-induced thrombocytopenia (19).

In our patient, the comprehensive etiologic study of her bicytopenia was negative. It ruled out alcoholism, hypothyroidism, cobalamin or folate´s deficiency, myelodysplastic syndromes, etc. These data and the temporal sequence (Figure 1) suggest that it could be a new adverse effect of Orlistat. The Naranjo adverse drug reaction probability scale ( 20 ) is widely used to assess the possibility of a new adverse effect. We applied it to our case, which was classified as a probable new adverse reaction. 

To our knowledge, there are no other reported cases of macrocytic anemia and thrombocytopenia due to Orlistat. The mechanisms involved are currently unknown.Furthermore, we do not know if it could be a dose-dependent phenomenon or not. 

Given the potential severity of the bicytopenia previously described, we would have to reconsider its free dispensing, or at least do studies to establish better such an adverse effect. 
